# Prognostic Nomogram for Disease-Specific Survival in Patients with Non-metastatic Ampullary Carcinoma After Surgery

**DOI:** 10.1245/s10434-018-07115-8

**Published:** 2019-01-18

**Authors:** Huang-bao Li, Feng-qing Zhao, Jun Zhou

**Affiliations:** Department of Hepatobiliary and Pancreatic Surgery, First Hospital of Jiaxing, First Affiliated Hospital of Jiaxing University, Jiaxing, Zhejiang People’s Republic of China

## Abstract

**Objective:**

The aim of this study was to establish and validate an individualized nomogram for predicting disease-specific survival (DSS) in patients with non-metastatic ampullary carcinoma after surgery.

**Methods:**

The nomogram was prepared using retrospective data from the Surveillance, Epidemiology, and End Results database, and included 2022 patients (training dataset: 1276; validation dataset: 746 patients) with non-metastatic ampullary carcinoma who were surgically treated between 2004 and 2014. Cox multivariate regression was performed to identify independent risk factors. The predictive accuracy was determined using the concordance index (C-index) and calibration curves. Results were validated internally using bootstrap resampling, and externally against the validation dataset.

**Results:**

The median follow-up for the training dataset was 25.5 months (range 1–143), the median survival time was 52 months [95% confidence interval (CI) 41.67–62.33], and the postoperative 1-, 3-, and 5-year DSS rates were 86.7%, 57.3%, and 47.2%, respectively. Univariate and multivariate regression analysis demonstrated that age, grade, tumor size, lymph node ratio, extension range, and histology were independent risk factors for DSS. The C-index of the internal validation dataset for predicting DSS was 0.70 (95% CI 0.68–0.72), which was superior to that of the American Joint Committee on Cancer staging, i.e. 0.64 (95% CI 0.62–0.66; *p* < 0.001). The 5-year DSS and median DSS time for the low-risk group were significantly greater than those for the high-risk group (*p* < 0.001).

**Conclusion:**

Our nomogram reliably and accurately predicted DSS in patients with non-metastatic ampullary carcinoma after surgery. This model may help clinicians in their decision making.

Ampullary carcinoma is the second most common periampullary malignancy.^1^ Because it is located at the end of the biliary tract, biliary obstruction occurs at the early stage of disease progression, showing typical symptoms, such as abdominal pain and jaundice. Therefore, it has a relatively high resection rate and better prognosis compared with other periampullary carcinomas.^2^ For non-metastatic ampullary carcinoma, surgical treatment, especially standard pancreatoduodenectomy, is the current standard treatment.^3^

The recently released 8th edition of the American Joint Committee on Cancer (AJCC) staging systems for ampullary carcinoma have introduced several improvements over previously used editions with respect to focus on tumor size and number of regional lymph node metastases.^4^ However, as a prognostic assessment model, many other factors affecting prognosis, such as tumor differentiation and age, are not considered. Moreover, it predicts outcomes in populations rather than in individuals. Therefore, the establishment of a more applicable prognostic evaluation model is necessary.

A nomogram is a simple, multivariate visualization prediction model that uses disease characteristics to determine individualized prognosis.^5^^–^10 Compared with traditional methods, nomograms are better predictors of individualized prognosis. However, to our knowledge, a nomogram model for ampullary carcinoma is currently non-existent. This study aimed to develop and validate a nomogram for individualized survival assessments in patients with ampullary carcinoma who were surgically treated.

## Materials and Methods

### Patient Datasets and Study Design

Data of 2022 patients with ampullary carcinoma who were surgically treated between 2004 and 2014 were extracted from the Surveillance, Epidemiology, and End Results (SEER) 18 Registries database. We randomly selected patients from six states (Atlanta, Detroit, Greater Georgia, Los Angeles, San Jose, and Seattle; *n* = 746) to comprise the validation dataset, and the remaining patients (*n* = 1276) were included in the training dataset.

All patients were diagnosed with ampullary carcinoma on the basis of histopathological examination. The analyzed variables included sex, age at diagnosis, race, marital status at diagnosis, grade, histology,  regional nodes examined (RNE), regional nodes positive (RNP), lymph node ratio (LNR, defined as the number of RNP divided by the RNE), tumor size, extension range, AJCC stage, cause-specific death classification, and survival in months. Follow-up ended in December 2015, and the primary endpoint was disease-specific survival (DSS), which was calculated as the interval between the date of diagnosis and the date of death from ampullary carcinoma.

The inclusion and exclusion criteria were consistent between the training and validation groups and are detailed as follows.

*Inclusion criteria* (a) Primary site of the tumor: (CS Schema v0204+, ampulla vater); (b) year of diagnosis: 2004–2015; and (c) no history of another malignant tumor (sequence number: one primary only; first malignant primary indicator: yes).

*Exclusion criteria:* (a) Non-operative treatment [RX Summ–Surg Prim Site (1998+): 0–27]; (b) age < 18 years; (c) tumor size unreported; (d) grade unreported; (e) survival time mismatch with the year of diagnosis; and (f) other variables that are unknown or are missing from the database.

Histologically, ampullary carcinoma is routinely divided into the pancreaticobiliary and intestinal subtypes,^11^,^12^ but the classification used in this study is based on the International Classification of Diseases for Oncology, Third Revision (ICD-O-3). Histological subtypes defined as ‘others’ were adenocarcinoma of the intestinal type (8144), adenocarcinoma in an adenomatous polyp (8210), adenocarcinoma with a mixed subtype (8255), papillary adenocarcinoma (8260), adenocarcinoma in a villous adenoma (8261), villous adenocarcinoma (8262), mucinous adenocarcinoma (8480), and signet ring cell carcinoma (8490). Extension range defined as ‘localized’ were tumors limited to the ampulla of Vater or extending to the sphincter of Oddi. Extension range defined as ‘adjacent organs or tissues’ were the tumor invades the following organs or tissues: blood vessel(s) [major]—hepatic artery, portal vein; gallbladder; hepatic flexure of the colon; lesser omentum; liver, including the porta hepatis; stomach; transverse colon; and peripancreatic soft tissue.

### Statistical Analysis

Summary statistics for the study population are presented as percentages or median values. The Mann–Whitney *U* test was used for continuous variables with a non-parametric distribution of patient data at baseline. The continuous variables were transformed into categorical variables to match the nomogram. The best cut-off points of continuous variables were identified using X-tile software (Rimm Laboratory, Yale School of Medicine, New Haven, CT, USA) for outcome-based optimization.^13^ Categorical variables were grouped according to clinical findings, and associations among categorical variables were tested using the Chi square test. Independent risk factors were screened by univariate analysis (log-rank) and forward stepwise Cox multivariate regression analysis using SPSS version 22.0 (IBM Corporation, Armonk, NY, USA). The DSS rate and median DSS were calculated using a life (actuarial) table method.

The nomogram was developed on the basis of independent risk factors and using the rms package in R version 3.5.0 (The R Project for Statistical Computing, Vienna, Austria). The predictive capacity of the nomogram was assessed using Harrell’s C-index (the concordance statistic, or C-statistic), which estimates the probability between the observed and predicted DSS.^14^ A random resampling procedure (bootstrapping) with 1000 resamples was used for internal validation, and the nomogram was externally validated with the validation dataset. The DSS derived from the developed nomogram, and the AJCC staging system, were compared using the rcorrp.cens (Hmisc) package in R and were assessed using the C-index.

The scores of each variable were calculated using the nomogramEx package in R. On the basis of the scores of each variable, the total DSS scores for each patient could be calculated. On the basis of the nomogram score, patients were then divided into low A-, low B-, moderate-, and high-risk groups. The 1-, 3-, and 5-year DSS rates and the median DSS time of each group were calculated, and the Kaplan–Meier survival curves were plotted. A *p* value < 0.05 was considered statistically significant.

## Results

### Patient Clinicopathologic Characteristics

In the training dataset, the ratio of men to women was 1.32:1 (725/551). The median patient age was 66 years, and adenocarcinoma accounted for 77.7% of the carcinomas. Most patients had early-stage AJCC tumors (I + II, 72.7%) and better differentiation (well + moderately differentiated, 66.1%). Table 1 presents all other patient clinicopathologic characteristics.

**Table 1:** Demographic and clinicopathologic characteristics of non-metastatic ampullary carcinoma patients after surgery

Demographic or characteristic	Training dataset		Validation dataset		*p* value
	No. of patients	%	No. of patients	%	
Sex					0.947
Male	725	56.8	425	57.0	
Female	551	43.2	321	43.0	
Age, years [median (IQR)]	66 (57–74)		64 (56–72)		0.021
Race					< 0.001
White	1017	79.7	534	71.6	
Black	65	5.1	86	11.5	
Others	194	15.2	126	16.9	
Marital status					0.177
Yes	825	64.7	460	61.7	
No	451	35.3	286	38.3	
Grade					0.407
Well-differentiated	134	10.5	86	11.5	
Moderately differentiated	710	55.6	386	51.7	
Poorly differentiated	423	33.2	268	35.9	
Undifferentiated	9	0.7	6	0.8	
Histology					0.229
Adenocarcinoma	991	77.7	583	78.2	
Others	285	22.3	143	21.8	
Regional nodes examined [median (IQR)]	13 (8–19)		14 (9–12)		0.017
Regional nodes positive [median (IQR)]	1 (0–2)		1 (0–3)		0.003
LNR [median (IQR)]	0.05 (0.00–0.20)		0.07 (0.00–0.22)		0.010
Tumor size, mm [median (IQR)]	21 (15–30)		22 (15–32)		0.336
Extension range					0.842
Localized	156	12.3	92	12.3	
Duodenal wall	364	28.5	203	27.2	
P/C/E	490	38.4	284	38.1	
Adjacent organs or tissues	266	20.8	167	21.4	
AJCC stage					0.468
IA	123	9.6	67	9.0	
IB	219	17.2	114	15.3	
IIA	169	13.2	88	11.8	
IIB	417	32.7	268	35.9	
III	348	27.3	209	28.0	

*P/C/E* pancreas, common bile duct, extrahepatic bile duct, *LNR* lymph node ratio, *AJCC* American Joint Committee on Cancer, *IQR* interquartile range

### Disease-Specific Survival (DSS) and Independent Risk Factors in the Training Dataset

The median follow-up was 25.5 months (range 1–143). The median survival time was 52 months [95% confidence interval (CI) 41.67–62.33], and the postoperative 1-, 3-, and 5-year DSS rates were 86.7%, 57.3%, and 47.2%, respectively. Univariate analysis demonstrated that age, grade, tumor size, RNP, LNR, extension range, and histology were risk factors for DSS, while multivariate analysis demonstrated that age, grade, tumor size, LNR, extension range, and histology were independent risk factors for DSS (Table 2). Sex, race, marital status, and RNE were not statistically significant in determining prognosis.

**Table 2:** Univariate and multivariate analysis of the training dataset and variable score

Variable	Score	Univariate analysis		Multivariate analysis		
		5-year survival (%)	*p* value	HR	95% CI	*p* value
Age, years			< 0.001			< 0.001
< 56	0	55.0		1		
56–73	32.07	48.8		1.24	0.98–1.57	0.071
> 73	64.14	37.4		1.89	1.46–2.44	< 0.001
Grade			< 0.001			< 0.001
Well differentiated	0	67.7		1		
Moderately differentiated	23.05	49.7		1.30	0.93–1.81	0.124
Poorly differentiated	46.10	35.4		1.72	1.22–2.42	0.002
Undifferentiated	69.15	–		0.22	0.03–1.60	0.134
Tumor size (mm)			< 0.001			0.030
≤ 14	0	62.7		1		
> 14	27.63	43.0		1.31	1.03–1.68	
RNP			< 0.001			
0	–	64.8				
1–2	–	34.6				
3–7	–	24.8				
> 7	–	22.5				
LNR			< 0.001			< 0.001
0	0	64.8		1		
≤ 0.21	50	34.6		1.87	1.50–2.32	< 0.001
> 0.21	100	24.1		2.70	2.16–3.39	< 0.001
Histology			< 0.001			0.006
Adenocarcinoma	29.98	44.4		1		
Others	0	56.7		0.74	0.59–0.92	
Extension range			< 0.001			< 0.001
Localized	0	67.9		1		
Duodenal wall	25.39	62.7		1.02	0.70–1.48	0.912
P/C/E	50.78	37.4		1.78	1.25–2.54	0.001
Adjacent O/T	76.18	31.6		1.82	1.24–2.67	0.002

*HR* hazard ratio, *CI* confidence interval, *RNP* regional nodes positive, *LNR* lymph node ratio, *P/C/E* pancreas, common bile duct, extrahepatic bile duct, *Adjacent O/T* adjacent organs or tissues

### Prognostic Nomogram for DSS

**Fig. 1:**
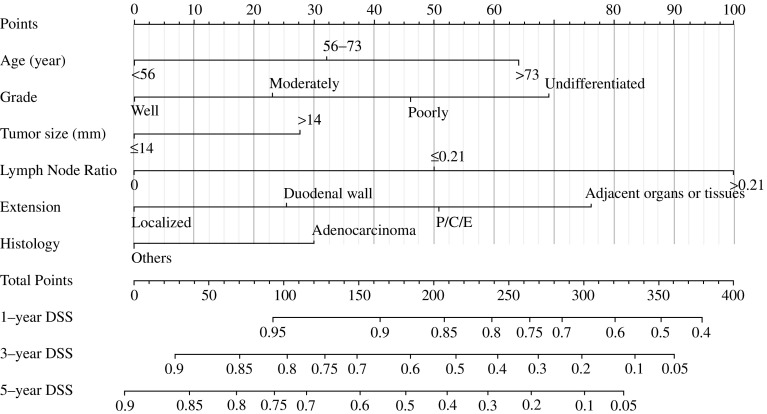
Nomogram for predicting the 1-, 3-, and 5-year DSS of patients with non-metastatic ampullary carcinoma after surgery. There were two methods to calculate the total scores. One method calculated the total scores based on the corresponding scores of each covariate on the ‘Points’ axis, and the other method calculated the total scores on the basis of the variable score provided in Table 2. The ‘Total Points’ axis was then used to determine the predicted probability. *P/C/E* pancreas, common bile duct, extrahepatic bile duct, *DSS* disease-specific survival

**Fig. 2:**
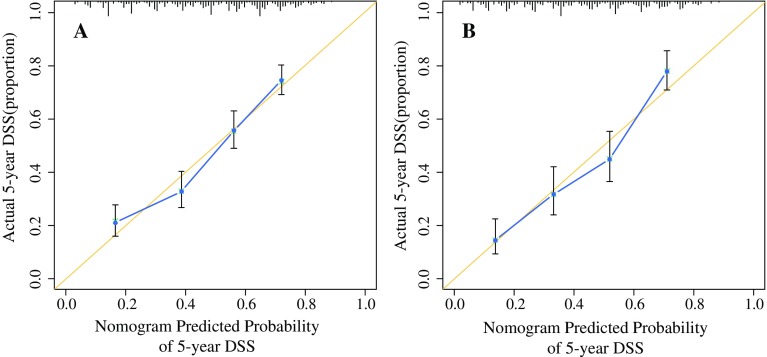
Calibration curves for predicting 5-year DSS for patients with non-metastatic ampullary carcinoma who were treated with surgery. The nomogram-predicted DSS probability is plotted on the x-axis, and the observed DSS is plotted on the y-axis. **a** Calibration curve for predicting patient survival at 5 years in the training dataset and **b** in the validation dataset. *DSS* disease-specific survival

Figure 1 shows the nomogram, which was generated using a Cox proportional hazards model that included all significant independent prognostic factors for DSS in the training dataset. The C-index of the internal validation for DSS prediction was 0.70 (95% CI 0.68–0.72), and the calibration curves for the probability of postoperative DSS at 5 years (Fig. 2a) suggested good consistency between the observed and predicted values. The nomogram showed superior performance compared with the AJCC staging system (C-index = 0.64, 95% CI 0.62–0.66; *p* < 0.001).

**Fig. 3:**
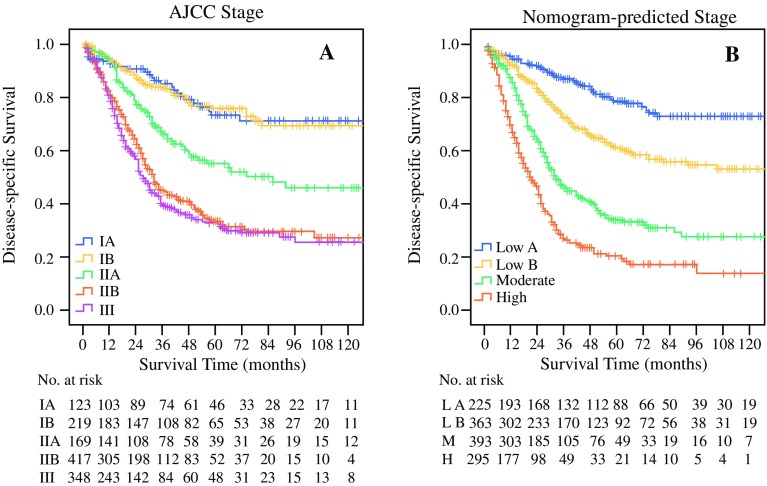
Kaplan–Meier survival curves for patients with non-metastatic ampullary carcinoma after surgery, according to **a** AJCC stages and **b** nomogram-based stages. The *p* value (< 0.001) was determined using the log-rank test. *AJCC* American Joint Committee on Cancer, *L A* low-risk A group, *L B* low-risk B group, *M* moderate-risk group, *H* high-risk group

Table 2 shows the scores of each variable. Patients with probability total scores < 120, 120–180, 180–240, and ≥ 240 were assigned to the low A-, low B-, moderate-, and high-risk groups, respectively. Figure 3 shows the Kaplan–Meier DSS curves based on AJCC stage (Fig. 3a) and separated by nomogram-based groupings (Fig. 3b). According to the prognosis curve predicted by different stages, discrimination of nomogram-predicted stages was better than that of AJCC stages. The 5-year DSS rates of the low A-, low B-, moderate-, and high-risk groups were 78.8%, 61.3%, 33.8%, and 20.1%, respectively. In addition, the median DSS time were 32 and 21 months for the moderate- and high-risk groups, respectively. The low-risk group had a 5-year survival rate of more than 50%, and had no median survival time. The 5-year DSS rates and median DSS time of patients were significantly higher in the low-risk group than in the high-risk group (*p* < 0.001).

### Validation of Predictive Accuracy of the Nomogram

In the validation dataset, the median DSS time was 43 months (95% CI 36.25–49.75), and the 1-, 3-, and 5-year DSS rates were 85.6%, 55.3%, and 42.2%, respectively. The C-index of the nomogram for DSS prediction was 0.70 (95% CI 0.67–0.73), and the calibration curve suggested good consistency between the observed and predicted 5-year DSS (Fig. 2b).

## Discussion

Accurate prognostication is essential to select perioperative therapy. However, to our knowledge, with the exception of the AJCC guidelines, there is no prognostic model for ampullary cancer, although a few studies have reported nomograms for periampullary carcinoma,^15^,^16^ including three different types, namely ampullary carcinoma, distal bile duct cancer, and pancreatic cancer, each with unique disease characteristics. They are all treated with pancreatoduodenectomy and have different prognoses.^2^ Therefore, these nomograms are unsuitable for ampullary carcinoma.

The present nomogram was constructed and validated using multivariate analysis, with age, grade, tumor size, LNR, extension range, and histology as independent risk factors. Although the 8th edition of the AJCC guidelines redefines the grouping of RNP and confirms its impact on prognosis,^4^ our study showed that LNR greatly impacts the nomogram, whereas the effect of the number of RNP is excluded from the Cox multivariate analysis. The result indicates that LNR plays an important role in prognosis assessment of ampullary carcinoma, a fact that has been demonstrated in other studies.^16^^–^18 LNR is incorporated into two variables—RNP and RNE; it not only considers the impact of RNP but also the impact of the total number of RNE on prognosis. Previous studies have shown that insufficient lymphatic dissection (RNEs) may result in the loss of positive lymph nodes (RNP) that have been metastasized, which might lead to residual metastases.^19^ Tumor grade is also an important factor affecting prognosis.^20^ In this study, we found that 66.1% of ampullary carcinoma patients were well and moderately differentiated, which may be the reason why the tumors had better prognoses. In addition, histological type is also a prognostic factor.^11^ Adenocarcinoma accounts for 77.7% of ampullary carcinomas (Table 2), but the prognosis is worse in comparison with that of other types. It is important to note that signet ring cell carcinoma often has a very poor prognosis,^21^ and although it is included in the ‘other’ group in this study, we do not recommend the use of this nomogram to assess the prognosis of the type.

The nomogram has the following advantages. First, the nomogram was superior to the current AJCC staging system in predicting DSS. This is not only reflected in its higher C-index value but also in the effect of differentiating the prognoses in different stages, as shown in Fig. 3. This is most likely because of the AJCC system only taking into account the tumor size, positive regional lymph nodes, and metastasis. However, age, LNR, differentiation grade, and extension of invasion were also independent risk factors for prognosis. Second, in practical terms, the variables used in the nomogram are easily obtained from patients with ampullary carcinoma who are treated surgically. By using the variable score, clinicians can immediately and accurately predict the prognosis and gain useful information regarding postoperative treatments. Third, clinical and pathological information of the nomogram is derived from the SEER database registered in 18 states of the US, which has features of multicenter clinical data; therefore, the results should be more applicable to the general population than if they were developed at a single institution.

When using this nomogram, we should pay attention to the following points. First, data were collected retrospectively. Second, the use of open access data from the SEER database did not include data on chemotherapy or the major comorbidities prevalent that could affect prognosis.^22^ Therefore, in the future, the prognostic disease-specific nomogram developed in this study should undergo external validation using an independent dataset.

## Conclusions

A prognostic disease-specific nomogram for patient survival in ampullary carcinoma after surgery was developed and validated. Clinicopathologic variables, including patient age, grade, tumor size, LNR, extension range, and histology were independent risk factors for postoperative prognosis. According to the score of the nomogram, because of the higher long-term survival rate, whether patients in the low-risk A group need excessive chemotherapy or radiotherapy needs further clinical observation.^23^ Moreover, patients in the high-risk group may have a poor prognosis and may need more active postoperative treatment and should be followed up more closely. Although this was a preliminary study, the nomogram was predictive of DSS in ampullary carcinoma patients who underwent surgery, and this model should be further evaluated in future clinical studies.
